# Antennal sensilla of two female anopheline sibling species with differing host ranges

**DOI:** 10.1186/1475-2875-5-26

**Published:** 2006-03-30

**Authors:** R Jason Pitts, Laurence J Zwiebel

**Affiliations:** 1 Department of Biological Sciences, Program in Developmental Biology, Institute for Chemical Biology and Center for Molecular Neuroscience. Vanderbilt University, Station B 351634Nashville, TN 37235-3582, USA

## Abstract

**Background:**

Volatile odors are important sensory inputs that shape the behaviour of insects, including agricultural pests and disease vectors. *Anopheles gambiae s.s. *is a highly anthropophilic mosquito and is the major vector for human malaria in sub-Sahara Africa, while *Anopheles quadriannulatus*, largely due to its zoophilic behaviour, is considered a non-vector species in the same region. Careful studies of olfaction in these sibling species may lead to insights about the mechanisms that drive host preference behaviour. In the present study, the external anatomy of the antenna, the principle olfactory organ in the female mosquito of both species, was examined as an initial step toward more detailed comparisons.

**Methods:**

Scanning electron and light microscopy were used to examine the antennae ultrastructures of adult female *An. gambiae s.s. *and *An. quadriannulatus*. Sensory structures, called sensilla, were categorized and counted; their distributions are reported here as well as densities calculated for each species.

**Results:**

Both *An. gambiae s.s. *and *An. quadriannulatus *bear five classes of sensilla on their antennae: chaetica (bristles), trichodea (hairs), basiconica (pegs), coeloconica (pitted pegs), and ampullacea (pegs in tubes). Female *An. quadriannulatus *antennae have approximately one-third more sensilla, and a proportionally larger surface area, than female *An. gambiae s.s. *antennae.

**Conclusion:**

The same types of sensilla are found on the antennae of both species. While *An. quadriannulatus *has greater numbers of each sensilla type, sensilla densities are very similar for each species, suggesting that other factors may be more important to such olfactory-driven behaviours as host preference.

## Background

Odors are the principle sensory signals that direct female mosquitoes to their preferred blood meal hosts [1,2]. Antennae of adult mosquitoes bear numerous sensory structures called sensilla, which are the physical sites of chemical detection. Within sensilla, olfactory signal transduction relies on odorant receptor proteins localized on the dendritic membranes of olfactory receptor neurons to initiate the events that ultimately lead to the perception of both the quality and the quantity of odors. Behavioural responses to volatile cues, including host finding by female mosquitoes, are critical components of vectorial capacity, the ability of an insect to transmit disease [2]. Two closely related mosquito sibling species, *An. gambiae s.s. *and *An. quadriannulatus*, display very different patterns of blood meal host preference. *An. gambiae s.s. *exhibits a high degree of anthropophily, while *An. quadriannulatus *exhibits strong zoophily [2]. Indeed, the strong preference for human blood meals by *An. gambiae s.s. *females is a major contributing factor to human malaria transmission, a disease that afflicts more than 200 million people and causes as many as 3 million deaths annually [3], while *An. quadriannulatus*, because of its preference for cattle, is not considered a malaria vector [4].

In order to understand the specifics of the attractiveness of humans to the mosquito, a major focus of this laboratory is the study of components in the olfactory signal transduction and coding process of *An. gambiae s.s. *Accordingly, comparisons of the peripheral and central olfactory events between behaviourally divergent sibling species, such as *An. gambiae s.s. *and *An. quadriannulatus*, should provide valuable insights into the mechanisms that determine their respective host preferences. Understanding the basic molecular events that underlie blood feeding may ultimately lead to the design of new ways to interfere with the human/mosquito interaction and thereby reduce the associated disease burden.

Antennal sensilla have previously been described for several mosquito species (for review see [5,6]), including some anophelines [7,8]. Apart from the large coeloconic sensilla, which are absent in the culicines [5], the types of sensilla found on mosquito antennae are generally well conserved, although large variations in the numbers of each type have been observed [5]. It is possible that variations in types or numbers of sensilla exist between *An. gambiae s.s. *and *An. quadriannulatus*, which may suggest areas of future investigation. As such, a comparative examination of the olfactory apparatus of *An. gambiae s.s. *and *An. quadriannulatus *as a prelude to future comparative molecular studies has been undertaken.

## Methods

### Mosquito rearing

*An. gambiae s.s. *(G3) and *An. quadriannulatus *(SANGQUA) were reared as described previously [9]. The G3 strain of *An. gambiae s.s. *was received from Dr. Mark Benedict at the Centers for Disease Control and Prevention and is described in more detail at: . *An. quadriannulatus *(SANGQUA) was the kind gift of Dr. Willem Takken (Wageningen Agricultural University, The Netherlands).

### Scanning electron microscopy (SEM)

Heads from 4- to 6-day-old adult *An. gambiae s.s. *or *An. quadriannulatus *were fixed with 4% paraformaldehyde, 0.1% Triton X-100 in phosphate buffered saline (PBS). Heads were then dehydrated; first in an ethanol series from 50% to 100% in 10% increments, followed by ethanol:hexamethyldisilazane (HMDS) at 75:25, 50:50, 25:75 and 0:100. HMDS was decanted and heads were dried in a fume hood. Heads were then glued onto aluminum pin mounts with colloidal silver paint and sputter coated for 30 seconds with gold-palladium. Samples were viewed using a Hitachi S-4200 scanning electron microscope and digital micrographs of each flagellomere were collected using Quartz PCI version 6.0 image acquisition software (Quartz Imaging Corp. Vancouver, B.C.).

### Light microscopy

Antennae from 4- to 6-day-old adult *An. gambiae s.s. *(G3 strain) or *An. quadriannulatus *were hand dissected from cold-anesthetized animals and placed in 25% sucrose, 0.1% Triton X-100 in water. These antennae were mounted on glass slides with a cover slip and sealed with clear enamel nail polish. Slides were stored at 4°C and observed under an Olympus BX-60 (Olympus America Inc. Melville, NY) microscope at 400× magnification. Photomicrographs were captured using an Olympus DP70 digital camera.

### Sensilla counts

Antennae were initially observed by SEM on both the dorsal and ventral aspects to look for any bias in sensilla arrangements. Thereafter, heads were mounted such that the antennae were most often observed from the lateral aspect, with some variation between individuals. Sensilla on each micrograph were classified by type and counted. Mean values for each sensillum type were calculated for 10 individuals per species and then multiplied by a factor of 2, assuming that only half the sensilla could be seen in each micrograph. For brightfield counting, all sensilla of a given type could be observed on each flagellomere by continuous focus adjustment through the specimen. Mean values for each type were calculated for 20 individuals. Standard deviations were calculated for each data set as described previously [10]. Standard errors (se) were then calculated by dividing the standard deviations by the square root of *n *[10]. Student's *t*-tests (two-tailed) were performed as described [10] to determine whether the mean number of each sensilla type differed significantly between species.

### Surface areas and densities

Flagellomeres 1–13 were viewed using brightfield optics, as described above, at 400× magnification. The length of each flagellomere was measured with an Olympus DP70 digital scale bar, and the width (diameter) was measured at the midpoint of the length (Figure 3A). The mean values of these measurements were calculated for 20 individuals per species. Standard errors (se) were calculated as described above. The surface area of a tube (length * diameter * pi) was used to estimate the surface area for each flagellomere. Mean surface areas were also calculated (n = 20 individuals per species). Standard errors (se) were calculated as described above. Student's *t*-tests (two-tailed) were performed to determine whether the mean surface areas differed significantly between species. Sensilla densities were calculated by dividing the mean number of sensilla by the total mean surface area where the particular type of sensilla was found.

**Figure 1:**
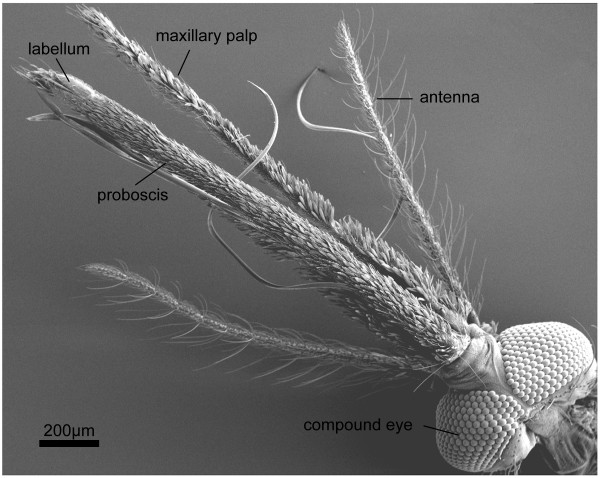
Female head (ventral view). Scanning electron micrograph showing the sensory appendages of an adult female *An. gambiae s.s. *Eyes, antennae, and maxillary palps occur in pairs, although the second palp is hidden below the proboscis in this micrograph. The proboscis is a single appendage that encloses the blood-feeding stylets, which appear as ribbon-like tentacles here. At the distal end of the proboscis is the labellum, or labellar lobes. The gross morphologies of the appendages are essentially identical in *An. quadriannulatus *females.

**Figure 2:**
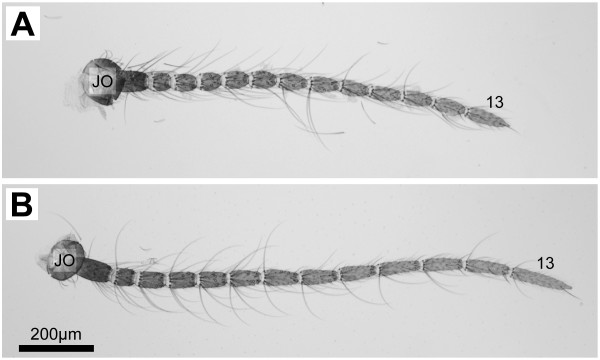
Female antennae. Brightfield images of single antenna from *An. gambiae s.s. *(A) and *An. quadriannulatus *(B) adult females. Flagellomeres are typically numbered 1–13 proceeding distally from the pedicel, or Johnston's Organ (**J.O.**). Flagellomere 13 is indicated for reference. Scale bar is the same for both images.

**Figure 3:**
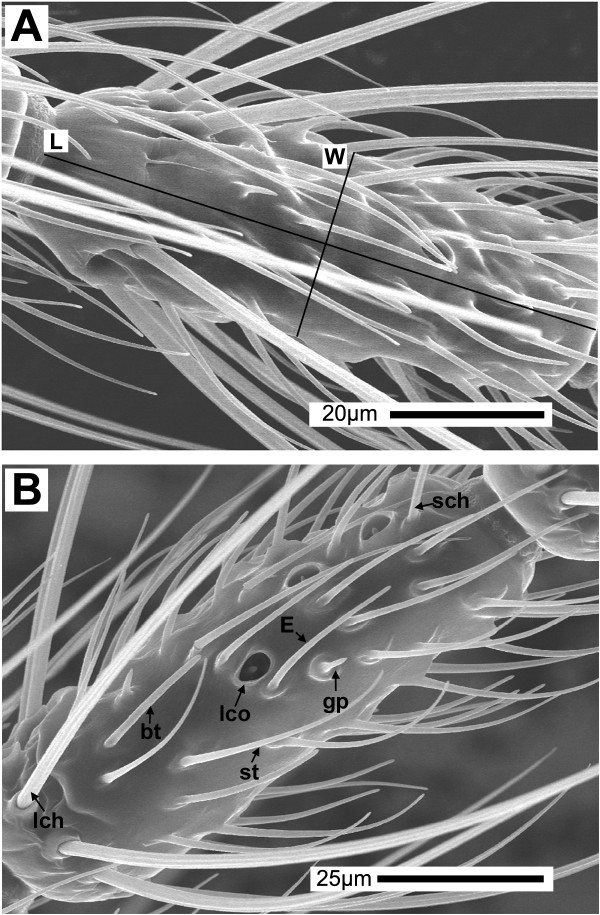
5th flagellomere. Scanning electron micrographs of flagellomere 5 from *An. gambiae s.s. *(A) and *An. quadriannulatus *(B) adult female antennae. (A) Length (**L**) and width (**W**) are measured as described in Materials and Methods. (B) Representative image showing the various sensilla types that are found on most flagellomeres: **lch **– large chaetica, **sch **– small chaetica, **st **– sharp trichoid, **bt **– blunt trichoid, **lco **– large coeloconic, **gp **– grooved peg, **E **– similar to type E trichoid. Note that the scale bars are different for each image.

**Figure 4:**
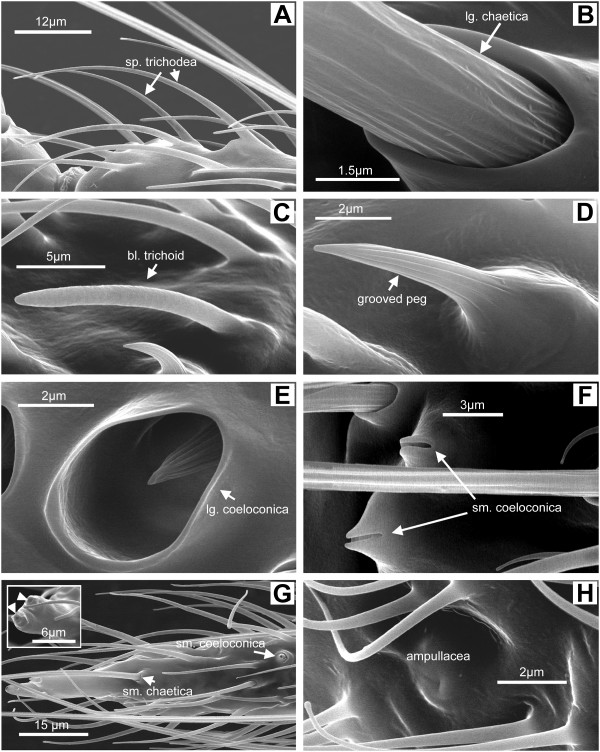
Sensilla types. Representative scanning electron micrographs showing sensilla types found on *An. gambiae s.s. *and *An. quadriannulatus *female antennae. External sensilla morphologies are indistinguishable in these species. (A) Sharp trichoid (hair) sensilla showing their smooth surfaces, socket-less bases, and tapered ends. (B) Base of a sensilla chaetica (bristle) with large socket. (C) Blunt trichoid (hair) sensillum. (D) Grooved peg (basiconic) sensillum. (E) Large coeloconic (pitted peg) sensillum with large cuticular opening and longitudinally grooved peg set deep within. (F) Small coeloconic (pitted peg) sensillum with small opening and peg not visible. (G) Tip of the 13th flagellomere showing small coeloconic sensilla at the distal end (inset arrowheads) and along the surface (arrow), as well as a single small chaetica (bristle). This sensilla arrangement is typical of both *An. gambiae s.s. *and *An. quadriannulatus *antennae. (H) Sensillum ampullaceum surrounded by microtrichia on the ventral surface of the first flagellomere.

**Figure 5:**
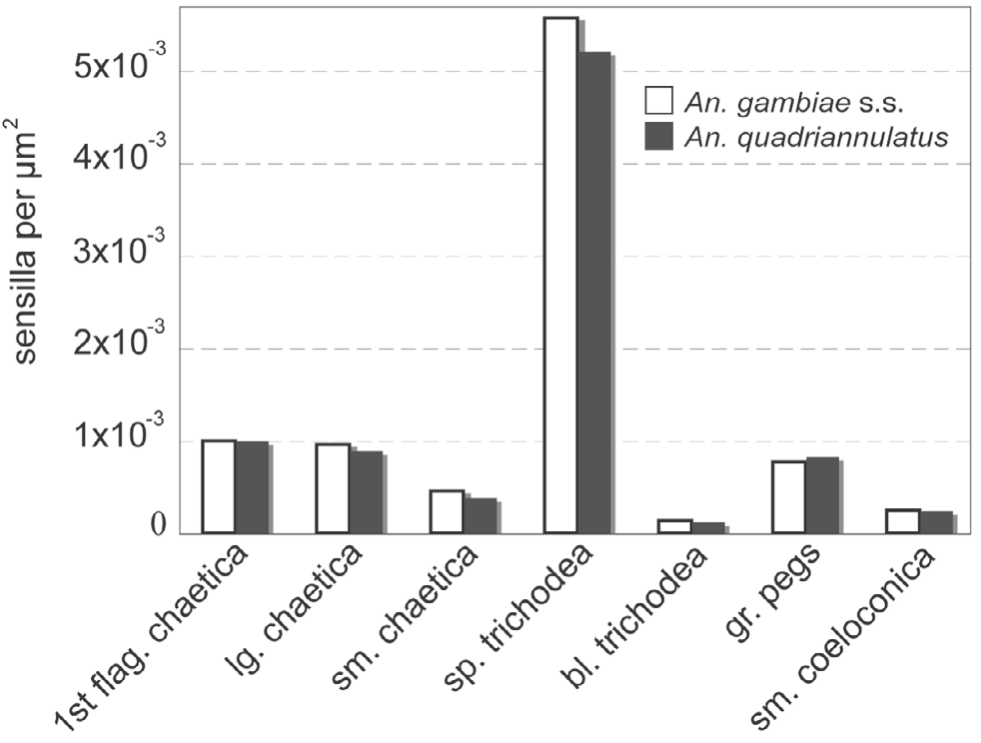
Sensilla densities. Histogram plot showing relative densities of sensilla types in *An. gambiae s.s. *and *An. quadriannulatus. *Y-axis is number of sensilla per μm^2 ^of surface area.

## Results

### General description

Like most dipterans, the heads of *An. gambiae s.s. *and *An. quadriannulatus *adults are equipped with three types of appendages – the antennae, maxillary palps, and proboscis (labellum) – each with associated chemosensory functions (Figure 1). The antennae and palps are sexually dimorphic in both species while the exterior of the proboscis is morphologically similar in both sexes.

In both species, each antenna is attached to the head by a structure called the scape, which is also an attachment point for some of the muscles that move the antennae [5]. Distal to the scape is the cup-shaped pedicel, which contains the Johnston's Organ (JO), and is the attachment point for flagellum (Figure 2). The flagellum is divided into 13 distinct flagellomeres, often referred to as segments (Figure 2). Female antennae house chemosensilla on all flagellomeres, while male antennae are populated by long fibrillae (bristles) on flagellomeres 1–11 with chemosensilla restricted to the distal two flagellomeres [5]. While the exact role of the fibrillae remains unclear, the specialized male antennae of sexually dimorphic species such as *An. gambiae s.l*. function as auditory sensors exquisitely tuned to the wing beat frequency of conspecific females for the purpose of locating a mate [11]. In both sexes the most distal, 13th, flagellomere is distinctive in that it tapers to a pointed tip, ending in a pair of small coeloconic sensilla (Figure 4G).

In addition to the input of the antennae, mosquitoes have two other sets of sensory head appendages. The maxillary palps are located latero-ventrally from the antennae and are divided into five segments in males and females of both species (Figure 1). The palps are a distinctive feature of female anophelines, being longer than the antennae, while in female culicines they are generally shorter than the antennae [5]. Numerous capitate pegs are located on the maxillary palps of both sexes and are the only sensillum type found there [5,6]. Capitate pegs have been shown to be sensitive to carbon dioxide and may therefore play a role in some aspects of host seeking [5,12].

The proboscis, or modified labium in mosquitoes, is also longer than the antennae and is the most ventral of the head appendages (Figure 1). At the distal end of the proboscis is the labellum, an organ divided into mirror-image labellar lobes that each house chemosensilla. While most sensilla on the labellum provide a gustatory function [5], there is a distinct subset, called type-2 (T2) chemosensilla, that expresses candidate odorant receptors [13] and has recently been shown to provide an olfactory function in *An. gambiae *(Kwon, Zwiebel et al., unpublished observations). Externally, male and female proboscises appear identical. However, males often lack or have highly modified forms of stylets enclosed within the proboscis [14].

Because of the major importance of the antennae on host seeking and other behaviours in female *An. gambiae s.s. *and *An. quadriannulatus*, this study focuses on a structural comparison of their antennae. The following are detailed descriptions of the types, numbers, distributions, and densities of female antennal sensilla.

### Sensilla chaetica

Sensilla chaetica are sturdy bristles that occur as two distinct subtypes – large and small. Both subtypes are set into sockets at their bases (Figure 4B) and end in sharply pointed tips; both the shape and the distribution of each are similar in *An. gambiae s.s. *and *An. quadriannulatus*. The large sensilla chaetica are arranged in a whorl on the basal end of each flagellomere 2–13 and distributed evenly around the circumference (Figure 3, 4B). *An. gambiae s.s. *and *An. quadriannulatus *displayed approximately 9 and 11 large sensilla chaetica per flagellomere, respectively. *An. gambiae *had an average of 105.6 per antenna, while *An. quadriannulatus *females had an average of 132 per antenna (Table 1).

**Table 1:** Types, numbers, and distributions of sensilla.

	*An. gambiae s.s.*			***An. quadriannulatus***					
	mean	se	dist	**mean**	**se**	**dist**	*p*	n	m

**1st flag. chaet.**	16.8	0.5	1	**23.3**	**0.9**	**1**	<0.001	20	b
**lg. chaet.**	105.6	1.9	2–13	**132.0**	**3.1**	**2–13**	<0.001	10	s
**sm. chaet.**	50.5	1.3	2–13	**57.5**	**2.0**	**2–13**	<0.01	20	b
**sp. trich.**	615.4	9.4	2–13	**772.4**	**21.6**	**2–13**	<0.001	10	s
**bl. trich.**	14.6	1.8	2–13	**19.2**	**2.3**	**2–12**	<0.20	10	s
**gr. peg**	79.4	3.5	3–13	**114.4**	**4.8**	**2–13**	<0.001	10	s
**lg. coel.**	21.6	0.4	1–9	**29.0**	**0.7**	**1–9**	<0.001	20	b
**sm. coel.**	~7	-	1,12,13	**~7**	**-**	**1,12,13**	-	20	b/s
**sum**	**910.9**			**1154.8**					

Summary of the average number of each sensilla type on the female antenna for each species. **mean **– average number of sensilla per antenna; **se **– standard error of the mean (+/-); **dist **– distribution of sensilla type by flagellomere number; **n **– individuals per species; **m **– survey method (**b **– brightfield, **s **– SEM); **sum **– sum of sensilla averages; ***p ***– significance level in two-tailed *t*-test comparing mean sensilla numbers.

Small sensilla chaetica were generally found on the dorsal surface and nearer the distal edge of flagellomeres 2–13 (Figure 3). Their numbers decreased slightly from the proximal to the distal flagellomeres. For example, *An. gambiae s.s. *was observed to have 5–7 on the first seven flagellomeres, 3–5 on the next five flagellomeres, and 1 or 2 on the 13th flagellomere (data not shown). Similarly, *An. quadriannulatus *had 6–8 on the first seven flagellomeres, 4–6 on the next five flagellomeres, and 1 or 2 on the 13th flagellomere. The number of small sensilla chaetica averaged 50.5 in *An. gambiae s.s. *and 57.5 in *An. quadriannulatus*.

The first flagellomere houses sensilla chaetica that are often difficult to classify as either large or small and therefore were counted as a single type in this analysis (Table 1). Generally, these chaetica were interspersed among numerous scales on the dorsal surface. Some of them are similar in appearance and placement to the small chaetica of flagellomeres 2–13 (Figure 3B). Also of note was a single chaeticum located near the tip of the 13th flagellum (Figure 4G). This sensillum is similar in structure to other small chaetica and was counted as such (Table 1). Averages of 16.8 and 23.3 chaetica were observed on the first flagellomeres in *An. gambiae s.s.* and *An. quadriannulatus*, respectively.

### Sensilla trichodea

The most numerous sensilla found along the flagellum of *An. gambiae s.s. *and *An. quadriannulatus *are the sensilla trichodea, which comprise two-thirds of all sensilla counted (Table 1). Two distinct types of sensilla trichodea were seen on the flagellum of both species and are most easily distinguished by their shapes, (Figures 3B, 4A, C). The sharp trichodea taper noticeably from base to tip, have a smooth surface without obvious grooves or ridges, and are not set into a socket (Figure 4A). Some researchers have divided the sensilla trichodea of mosquitoes into separate sub-classes based on lengths, shapes, and wall thicknesses [5], with as many as five sub-classes described for *An. stephensi *[15]. While great variation in the lengths of the sharp trichodea were observed in this study, all sensilla of this type were of a similar shape and were therefore counted together (Table 1). Of note are very short, sharp trichoid sensilla (Figure 3B) that were seen in low numbers and may be the equivalent of the type E trichoid sensilla described for *An. stephensi *[15]. The numbers of sharp sensilla trichodea increased dramatically from proximal to distal flagellomeres in both species. Usually zero, but occasionally a few, sharp trichoid sensilla were found on the first flagellomere. *An. gambiae s.s. *was shown to house approximately 13 on flagellomere 2, 60 on flagellomere 7, and 70 on flagellomere 13, while *An. quadriannulatus *housed about 24, 75, and 90 for the same segments (data not shown). The sharp sensilla trichodea seemed to be randomly distributed around the circumference of each flagellomere 2–13. *An. gambiae s.s. *females had, on average, about 615 trichoid sensilla per antenna, and *An. quadriannulatus *females had about 770 per antenna in this study.

Blunt trichodea also lack grooves or ridges, but are distinct from the sharp trichodea in that they do not taper sharply, ending instead in a rounded tip that is nearly as wide as the base (Figure 4C). These hairs were also apparently more uniform in length than the sharp trichodea. In *An. gambiae s.s. *and *An. quadriannulatus*, blunt sensilla trichodea were the least numerous class of sensilla quantified in this study (Table 1). They were found in small numbers on flagellomeres 2–13 but were rarely seen on the distal 4 flagellomeres. Each antennae of *An. gambiae s.s. *and *An. quadriannulatus *females was found to house 15–20 blunt sensilla trichodea on average (Table 1).

### Sensilla basiconica

Another distinct class of antennal chemosensory structures are the sensilla basiconica, or grooved pegs, which were found on the flagella of both *An. gambiae s.s. *and *An. quadriannulatus*. They closely resemble the sensilla basiconica of *Aedes aegypti *and other culicines [5], which appear externally as thorn-shaped hairs with 10–12 grooves running along their surfaces and are raised on small prominences that lack sockets (Figures 3B & 4D). Grooved pegs were observed on flagellomeres 3–13 in both species, but also appeared infrequently on the second flagellomere in *An. quadriannulatus *(Table 1). Their frequency increased distally along the flagellum with nearly half of them occurring on the last three flagellomeres of both species. No surface distribution bias (i.e., dorsal, ventral, etc.) was observed in either species, which agreed with a previous finding that surface pegs occur on all aspects of the flagellar surface [16].

Grooved peg sensilla looked similar in the SEMs of *An. gambiae s.s. *and *An. quadriannulatus *so that no subclass distinctions were made in this study. However, a previous study identified two distinct subclasses in *An. stephensi *based on their number of external grooves, wall structures, and number of innervating neurons [16]. It is therefore possible that grooved peg subtypes exist in *An. gambiae s.s. *and *An. quadriannulatus*. Along the *An. gambiae s.s. *antenna, 79.4 grooved pegs were found, on average, and 114.4 were found along the *An. quadriannulatus *antenna (Table 1).

### Sensilla coeloconica

Sensilla coeloconica are small, thick-walled sensilla that occur in large and small forms in the anophelines [5]. Large sensilla coeloconica are commonly called pitted pegs and are absent in the culicines [5,6]. As the common name implies, pitted pegs appeared as round openings in the cuticle with single peg-shaped setae projecting from within and parallel to the walls of the pit (Figures 3B &4E). Their tips often projected to just below the external rim of the pit (Figure 4E). Like the basiconic sensilla, the pegs of large coeloconic sensilla were grooved length-wise, but generally had more grooves than the former (compare Figures 4D and 4E). These sensilla were always observed on flagellomeres 1–7 in both species, with their greatest numbers occurring on flagellomeres 2–5. In *An. gambiae s.s.*, a single large coeloconic sensillum was located on either flagellomere 8 or 9 in only 30% of antennae, and on both flagellomeres in just 10% of antennae. However, in *An. quadriannulatus*, a single large coeloconic sensillum was found on either flagellomere 8 or 9 in 90% of antennae, and on both in 80% of antennae. *An. gambiae s.s. *females had an average of 21.6 large coeloconica per antenna and those of *An. quadriannulatus *had an average of 29 (Table 1).

Small sensilla coeloconica also have a peg set into the bottom of a pit [5]. These sensilla had a much smaller cuticular opening than the large coeloconica, and the peg did not protrude from the opening enough to be seen in SEMs. The distal tip of the 13th flagellomere in both *An. gambiae s.s. *and *An. quadriannulatus *ended in 2 (or rarely 3) small sensilla coeloconica (Figure 4G inset). Furthermore, 3 small coeloconica were usually observed on the distal edge of the first flagellomere (Figure 4F) and 1 on flagellomeres 12 and 13 for both *An. gambiae s.s. *and *An. quadriannulatus *(Figure 4G). Although not formally quantified in this study, both species were observed to have about 7 small coeloconica per antenna (Table 1). A previous study described these sensilla as "campaniform" using the light microscope to examine them [7]. A close examination of these sensilla using the scanning electron microscope revealed that they were in fact not dome-like campaniform sensilla, but rather appeared as volcano-like structures with an opening at the peak. Small coeloconica were not observed on segment 2 in *An. gambiae s.s. *or *An. quadriannulatus*, in contrast to those described previously in *An. stephensi *[17].

### Sensilla ampullacea

Sensilla ampullacea are small, thick-walled peg sensilla set at the bottom of a tube, the external opening of which appears as a very small aperture on the cuticular surface[5]. Unlike coeloconic sensilla, the pegs project perpendicularly to the tube walls [17]. They were observed in small numbers on the ventral surface of the first flagellomere in both *An. gambiae s.s. *and *An. quadriannulatus *(Figure 4H). Their small size and location made the ampullaceae the most difficult to discern as they were often obscured by the numerous non-innervated hairs, or microtrichia, which surrounded them (Figure 4H). Therefore, accurate counts were not possible using the survey method presented here. In this study, no ampullacea were seen on flagellomeres 2–13, although their existence could not be excluded. The occurrence of eight ampullaceae on the medioventral aspect of the first flagellomere and one on the second flagellomere has been described for *An. stephensi *[17].

### Surface areas and densities

While in the process of observing and counting sensilla, it became apparent that *An. quadriannulatus *antennae were larger than those of *An. gambiae s.s. *(Figure 2). This size disparity could account for the differences in the numbers of sensilla between the species. Therefore, attempts to describe an average antenna size for female *An. gambiae *s.s. and *An. quadriannulatus *were carried out. To do this, flagellomere lengths and widths (Figure 3A) were measured and used to calculate mean surface areas. In every case, *An. quadriannulatus *flagellomeres seemed to have a greater surface area than *An. gambiae s.s. *flagellomeres (Table 2).

**Table 2:** Flagellomere dimensions and surface areas.

	***An. gambiae s.s***						***An. quadriannulatus***						
**flag**	L	se	W	se	SA	se	**L**	**se**	**W**	**se**	**SA**	**se**	*p*

**1**	112.3	1.8	47.9	1.3	16848	467	**127.3**	**1.8**	**57.9**	**0.7**	**23176**	**565**	<0.001
**2**	57.0	0.9	40.1	0.9	7154	136	**69.4**	**1.0**	**47.2**	**0.7**	**10301**	**244**	<0.001
**3**	69.3	0.9	39.4	1.0	8545	183	**80.1**	**1.1**	**44.0**	**0.7**	**11088**	**291**	<0.001
**4**	71.9	0.6	38.4	1.0	8659	233	**85.0**	**1.0**	**42.8**	**0.7**	**11440**	**253**	**<0.001**
**5**	76.9	0.9	38.4	0.9	9260	178	**89.4**	**1.1**	**43.2**	**0.7**	**12147**	**293**	**<0.001**
**6**	81.3	0.9	37.3	0.9	9506	206	**94.4**	**1.2**	**42.3**	**0.7**	**12553**	**258**	<0.001
**7**	82.8	3.0	37.0	0.9	9612	223	**100.1**	**1.1**	**41.9**	**0.6**	**13163**	**256**	<0.001
**8**	87.2	0.7	34.7	1.0	9504	311	**104.1**	**1.4**	**37.5**	**0.6**	**12259**	**192**	<0.001
**9**	86.7	0.9	34.2	1.0	9321	318	**104.1**	**1.3**	**37.8**	**0.7**	**12369**	**283**	<0.001
**10**	89.7	4.9	31.8	1.0	8972	355	**112.3**	**1.4**	**33.5**	**0.9**	**11788**	**290**	<0.001
**11**	89.6	1.3	32.1	1.1	9041	348	**115.6**	**1.7**	**33.5**	**0.6**	**12160**	**256**	<0.001
**12**	96.6	1.5	31.0	1.1	9416	371	**121.7**	**1.7**	**32.2**	**0.6**	**12276**	**207**	<0.001
**13**	134.7	1.7	27.2	1.0	11506	468	**164.1**	**1.7**	**32.2**	**0.6**	**16552**	**309**	<0.001

Summary of the flagellomere dimensions of the female antenna for each species. **flag **– flagellomere number; **L **– average length (μm); **W **– average width (μm); **se **– standard error of the mean (+/-); **SA **– mean surface area per flagellomere (μm^2^); ***p ***– significance level in two-tailed *t*-test comparing mean surface areas. n = 20 individuals per species.

Finally, the mean numbers of sensilla and mean surface areas were used to estimate sensilla densities. For each sensilla type, the mean number of sensilla was divided by the sum of the surface areas of the flagellomeres where each was distributed. For example, to estimate the density of sharp trichodea in *An. gambiae s.s.*, 615.4 (Table 1) was divided by the sum of the mean surface areas of flagellomeres 2–13, 110498μm^2^, to arrive at a density of 5.6 × 10^-3 ^sensilla/μm^2 ^(Figure 5). Within both species, the sharp trichodea were found in the highest density, while the blunt trichodea were found in the lowest density (Figure 5). Each sensilla type was found to be similarly dense in *An. gambiae s.s. *and *An. quadriannulatus *(Figure 5).

## Discussion

Five classes of sensilla have previously been described on the flagellomeres of mosquitoes [5]. Each of these classes were observed on the antennae of *An. gambiae s.s. *and *An. quadriannulatus*. The distribution pattern of each sensilla type was highly conserved among individuals within a single species and was also very well conserved between *An. gambiae s.s. *and *An. quadriannulatus*. However, the average numbers of sensilla seemed to differ between these two species. In fact student's *t*-tests confirmed that the interspecific mean values for nearly all sensilla types were significantly different (Table 1). The lone exception was the blunt trichodea where the *t*-test returned a low significance value (*p *< 0.20, Table 1). The mean number of blunt trichoid sensilla may in fact be different, but their rarity may exaggerate any bias that is inherent in the method used here to count them, since SEMs offer a view of only one side of the flagellomere.

The calculated mean numbers of *An. gambiae s.s. *sensilla are in general agreement with previous work [7], with the exception of the number of large coeloconic sensilla. Ismail reported 33 per antenna, distributed on flagellomeres 1–9, including 2 per flagellomeres 8 and 9 [7]. In contrast, an average of only 21.6 per antenna was found in this study, being infrequently observed on flagellomeres 8 and 9. It is reasonable to suggest that variation between the laboratory strains of *An. gambiae *used in these studies may underlie these differences. Indeed, great variation in the numbers of large coeloconic sensilla has been observed in wild populations of *An. gambiae s.l*. [8].

Significantly greater mean surface areas of antennal flagellomeres were observed in *An. quadriannulatus *than in *An. gambiae s.s. *(Table 2). This difference accounts for the disparity in the numbers of sensilla, as it appears that the densities of antennal sensilla are nearly the same in both species (Figure 5). This conclusion could not be supported by a statistical comparison of sensilla densities because the mean sensilla numbers and mean surface areas from which the densities were derived were calculated using data collected from different groups of individuals. Nonetheless, the conservation of sensilla types and densities may indicate conservation of the underlying developmental program in female antennae. Thus it seems unlikely that either peripheral structures or their organizational patterns contribute to behavioural differences in these species. Furthermore, it is likely that adult body size differences account for the antennal surface area difference and, thus, the difference in sensilla numbers. A commonly used comparative measurement of body size in mosquitoes is wing length [18]. A derivation of this technique, measuring both the wing length and width, was used to calculate the wing surface area for both species. Not surprisingly, *An. quadriannulatus *wings were found to be about 30% greater in surface area than those of *An. gambiae s.s.*, a difference that is nearly the same in magnitude as their antennal surface area differences. One possible explanation for the adult size variation between the two species is larval density during rearing, a factor that is known to produce a larger adult size in *An. gambiae *laboratory-reared populations [19]. While the rearing conditions were similar for both species, *An. quadriannulatus *generally produced fewer offspring and, therefore, were kept at lower densities during the larval stage.

Of the sensilla types, sensilla trichodea have been found in the greatest abundance on the mosquito flagellum [5]. In this study there were approximately 7-fold more trichoid sensilla than grooved pegs, the second most abundant class of chemosensory sensilla in both *An. gambiae s.s. *and *An. quadriannulatus *(Table 1, Figure 5). Previous studies with *An. stephensi *[15] and *Ae. aegypti *[20] indicated that nearly all trichoid sensilla were innervated by two neurons. If we assume a similar situation in *An. gambiae s.s. *and *An. quadriannulatus*, then these flagella must house at least 1200 and 1500 neurons within their trichoid sensilla populations, respectively. Sensilla trichodea on female *An. gambiae *have been shown to respond to carboxylic acids [21] and carboxylic acids are also attractive to *An. gambiae *in behavioural studies [22]. Similarly, electrophysiological [23] and behavioural [24] studies in *Ae. aegypti *have suggested that trichoid sensilla house olfactory receptor neurons. It is therefore possible that trichoid sensilla neurons represent the major portion of the olfactory receptor repertoire on mosquito antennae and, as such, deserve significant attention in future studies, particularly those designed to compare behavioral and physiological differences between *An. gambiae s.s. *and *An. quadriannulatus*. Notably, some variations in the sensitivities of trichoid sensilla have been observed between anthropophilic and zoophilic anophelines [25].

Grooved pegs are the second most abundant class of sensilla on the antennae of *An. gambiae s.s. *and *An. quadriannulatus *(Table 1). Their potential importance in host seeking behaviours has been suggested by studies in various mosquito species that demonstrate the sensitivity of grooved pegs to human sweat components, including ammonia [26,27] and lactic acid [28]. Moreover, behavioural studies also implicate these odors, especially ammonia, in host attractiveness [29], while lactic acid seems to have a synergistic effect in combination with other odors [30-32]. Importantly the responses of grooved peg sensilla to a number of odors, including ammonia, were very similar in *An. gambiae s.s. *and *An. quadriannulatus *[33]. The authors conclude that trichoid sensilla and grooved peg sensilla respond to overlapping sets of host odors and that these sensilla may therefore be part of a generalistic host sensing mechanism with host specific information being derived from the combined information of these inputs [33]. The current study provides an important morphological framework for continued comparative analyses.

There are at least six species in the *An. gambiae s.l*. complex, some living sympatrically, which seem to have diverged very recently [34]. Furthermore, there is evidence supporting the existence of a second species of *An. quadriannulatus*, and reason to believe that more *An. gambiae s.l*. species may yet be identified [35]. While the close relatedness of *An. gambiae s.s. *and *An. quadriannulatus *may imply olfactory conservation at the level of peripheral sense organs, there is a rational basis to evaluate this assumption considering both the great divergence in their host preferences and the observed diversity in mosquito peripheral organs [5]. Clearly, in this study, we have established that the antennae ultrastructures of *An. gambiae s.s. *and *An. quadriannulatus *are extremely similar and are thus unlikely to contribute to the behavioural differences that underlie their characteristic anthropophily and zoophily, respectively. Therefore, differences such as odorant receptor sensitivities, signal transduction components, internal morphologies (i.e., neuronal architecture and projections), or environmental factors are more likely to contribute to the host preference divergence of *An. gambiae s.s. *and *An. quadriannulatus*. A recent comparison of candidate olfactory receptor genes in *An. gambiae s.s. *and *An. quadriannulatus *showed an extremely high level of conservation between their amino acid sequences (Bohbot and Zwiebel, unpublished observations). This study provides an essential foundation for these and other future comparative analyses that will focus on the molecular genetics and physiology of olfaction in these two species.

## Conclusion

The antennae of adult female *An. gambiae s.s. *and *An. quadriannulatus *mosquitoes carry the same morphological types of sensilla and the densities of each type are effectively equal between the two species. Therefore, the lack of specialization at the gross morphological level of the antennae implies that other factors are more likely to account for the olfactory-driven host preference difference between *An. gambiae s.s. *and *An. quadriannulatus*. This study establishes a foundation for future neurological, physiological, and molecular comparative studies aimed at elucidating potential differences in olfaction between these sibling species.

## Authors' contributions

RJP and LJZ conceived and designed the study. RJP carried out all microscopy and analysis (with some assistance from undergraduate researchers as listed in the acknowledgements) and drafted the manuscript. Both authors read and approved the final manuscript.
